# Screening of Profitable Chrysanthemums for the Phytoremediation of Cadmium-Contaminated Soils

**DOI:** 10.3390/toxics13050360

**Published:** 2025-04-30

**Authors:** Xinzhe Lu, Yanfang Chen, Jinqiu Song, Jiayu Bao, Chunzheng Dai, Rui Sun, Jiacheng Liu, Chenjiang Jin, Nanchong Zhong, Chunlei Huang, Kokyo Oh

**Affiliations:** 1 Zhejiang Institute of Geosciences, Hangzhou 310007, China; 15268511668@126.com (Y.C.); nearysr@163.com (R.S.); 2 Technology Innovation Center of Ecological Evaluation and Remediation of Agricultural Land in Plain Area, MNR, Hangzhou 310007, China; 13069826196@163.com (J.B.); ljc13755921635@163.com (J.L.); 3 Zhejiang Zhezhong Geological Engineering Investigation Institute Co., Ltd., Jinhua 321001, China; sjq_840311@163.com (J.S.); 13757993951@139.com (C.D.); rockcold87@hotmail.com (N.Z.); 4 School of Earth Sciences and Resources, China University of Geosciences, Beijing 100083, China; 5 Zhejiang Geology and Mineral Technology Co., Ltd., Hangzhou 311305, China; flcl@163.com; 6 Center for Environmental Science in Saitama, 914 Kamitanadare, Kazo City 347-0115, Japan; o.kokyo@pref.saitama.lg.jp

**Keywords:** phytoremediation, cadmium remove, soil contamination, chrysanthemum cultivars

## Abstract

To explore the phytoremediation effect of ornamental chrysanthemums on cadmium (Cd)-contaminated farmland soil, a 2-year field trial was conducted on 23 chrysanthemum cultivars in Cd-contaminated soil in Zhejiang Province, China. The biomass yields, Cd content of the plants, Cd enrichment coefficient, and remediation efficiency were evaluated. The aboveground biomass of the tested chrysanthemums was 67.10–166.08 g/plant, the aboveground Cd content was 1.97–5.92 mg kg^−1^, and the Cd enrichment coefficient was 2.98–9.84. In a screening test of twenty-three chrysanthemum cultivars, six cultivars, such as marigolds, were characterized by high cadmium accumulation, with the average cadmium accumulation of chrysanthemums exceeding 0.6 mg per plant, and the remediation of rhizosphere-contaminated soils took only 4–5 years. Fourteen chrysanthemum cultivars have good multiple-cropping characteristics, and five multiple-cropping chrysanthemum cultivars, such as QX-yz, have high heavy metal tolerance. The multiple-cropping JL-yg cultivars with higher Cd accumulation could be recommended for the remediation of Cd-contaminated farmland. The application of bamboo vinegar to the chrysanthemum rhizosphere effectively promoted Cd absorption. After estimating the economic benefits of artificially planting five dominant varieties of chrysanthemums for polluted farmland remediation, it is concluded that the annual income of a worker can be slightly higher than the average annual income level of local residents.

## 1. Introduction

Heavy metal cadmium (Cd) is among the most serious and common pollutants in the soil. Soil pollution with heavy metals can harm animals and humans by getting into the food chain [1,2]. Currently, heavy metal-contaminated soil can be remediated by physical, chemical, or ecological methods (e.g., phytostabilization, microbial remediation, and biochar amendment) [3,4,5,6,7]. Phytoremediation employs hyperaccumulators to extract and concentrate soil heavy metals [8,9,10,11]. While phytoremediation can be cost effective when integrated with cash crops, traditional monoculture hyperaccumulators often lack economic returns [12]. In Chinese culture, chrysanthemum is known as one of the ‘Four Gentlemen’ and is widely used for ornamental purposes. A suburban area of Shanghai has been restored for safe use (Cd < 0.3 mg/kg, Zn < 150 mg/kg through the removal of excessive Cd and Zn by planting the chrysanthemum ‘June White’ [13,14]. Chrysanthemums have been found to have the advantages of easy cultivation, high survival rate, and heavy metal tolerance, and they have economic benefits as ornamental flowers, chrysanthemum tea, Chinese medicinal materials, etc. We consider the selection of chrysanthemums that bring economic benefits while achieving the reuse of contaminated land. The selection criteria are (1) BCF > 1.5; (2) market price ≥ RMB50/kg; and (3) biomass ≥ 3 tons/hectare.

In addition, some researchers are exploring the possibility of ethylenediamine tetraacetic acid (EDTA) [15], ethylenediamine succinic acid (EDDS) [16], diethyl triacetic acid (NTA) [17], and other synthetic chelating agents and low-molecular-weight organic acids to improve phytoremediation efficiency. Although a synthetic chelating agent can induce the desorption of heavy metal ions in the soil, such as Cd^2+^ and Pb^2+^, it also creates the potential environmental problem of complexing and making other mineral elements in the soil unavailable, such as Ca^2+^ and Mg^2+^ [18], which is not conducive to the recovery and subsequent reuse of the remediated soil. Even at low doses, EDTA can inhibit the growth of rhizosphere microorganisms [19]. The biodegradable chelating agent NTA is not recommended for soil remediation as a result of its potential carcinogenicity. Citric acid can effectively promote plant absorption and the accumulation of Cd without affecting the normal growth of plants [20]. However, compared with an artificial chelating agent, a natural organic acid is more expensive, so there remain considerable limitations in the practical application and promotion of organic acids under the premise of limited remedial efficiency. The main component of bamboo vinegar is natural organic acid, which can increase the content of exchangeable heavy metals in the soil by forming soluble complexes with heavy metals or lowering the pH value of the soil to improve the efficiency of the plant’s root system in absorbing heavy metals such as Cd^2+^, Pb^2+^, and other cations with similar ionic radii without affecting the normal growth of the plant. In addition, bamboo vinegar can be obtained in the process of bamboo charcoal production, and bamboo charcoal firing and product development have been industrialized.

In this study, heavy metal-contaminated farmland soil in a typical industrial area was selected for remediation. Chrysanthemum cultivars that have strong heavy metal accumulation, high tolerance characteristics, high biomass production, and additional economic benefits were selected for field trials. Testing the effectiveness of the chelating agents was combined with phytoremediation.

## 2. Materials and Methods

### 2.1. Study Site

This study was conducted in a 1 ha area of Cd-contaminated farmland (28°56′30″–28°56′50″ N, 120°07′30″–120°07′40″ E) located in a hardware-processing industrial town in Zhejiang Province, eastern China, with a subtropical monsoon climate. In 2020, a survey found that the Cd content in farmland soil from the study area exceeded the permitted limit of the Chinese national standard ‘Soil Environmental Quality Standard for Soil Pollution Risk Control of Agricultural Land (Trial)’ [13]. The content of Cd in soil is 0.32 mg kg^−1^, and the exceeding rate of rice samples is nearly 30%. Cd pollution was mainly caused by sewage leaching from surrounding hardware-processing enterprises.

### 2.2. Experiment Design and Treatments

**Figure 1:**
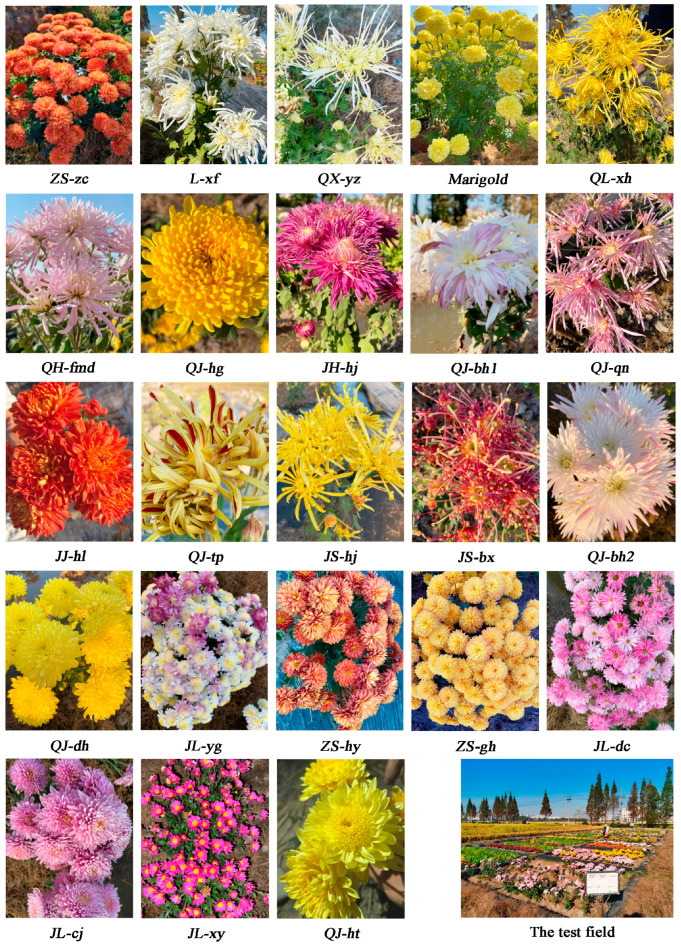
Chrysanthemum cultivars used in this study.

In the trial field, we established 23 test plots, and each plot covered an area of 12 m^2^. The ridges with 0.3 m (height) multiplied by 0.3 m (width) were wrapped with film up to the bottom of the plow and encircled the test plots. Each plot had a one-way drainage ditch installed. A total of 23 cultivars of chrysanthemums were planted in a nearby plant factory for the experiment. Seedlings were grown between 10 March and 10 April 2021, and the best-growing cuttings were selected and transplanted to the experimental field. Each plot had only one type of chrysanthemum transplanted, and we planted the chrysanthemums in three rows with a spacing of 20 cm. The growth cycle of the chrysanthemum was from 10 April to 10 October. After the flowering period, all the chrysanthemums for the test were pruned, keeping a stem length of 10–12 cm and 3–4 chrysanthemum leaves, and the transplanting of chrysanthemums to the test field was not repeated in 2022. The 2022 campaign focused on multi-cropping effects rather than mere repetition, with sequential sampling conducted at the flowering stages of each crop cycle. Photographs of the test area and chrysanthemum cultivars are shown in Figure 1, and the full cultivar names are shown in Table 1. All the chrysanthemums purchased from Chrysanthemum Base of Nanjing Agricultural University, Jiangsu Province, China. 

In order to improve the chrysanthemum remediation efficiency of contaminated soil, the *Chrysanthemum × morifolium* ‘Qianxiuyinzhen’ cultivar was selected to perform heavy metal chelator-coupled phytoremediation experiments based on the results of the 2021 experiment. There were five groups of treatments in the experiment, and each group of treatments had three experimental plots. The method and dosage of the chelating agent are shown in Table 2, all the chelating agents purchased from Jiangsu Fertilizer Industry Co., LTD, Jiangsu Province, China. The chelating agent was sprayed between the roots of chrysanthemums in small quantities several times during the application. 

### 2.3. Sampling and Experiment Analysis

Three groups of chrysanthemum plants and their rhizosphere soil were randomly taken from each plot during their flowering in 2021, and flower, stalk, and rhizosphere soil of multiple-crop chrysanthemum cultivars were collected in 2022. All samples were collected from the plant rhizosphere soil with a thickness of 0–20 cm, air-dried, ground, and passed through a 2 mm sieve before the test. The flower and stalk were dried, weighed, and pulverized for the test.

Cd content in soil and chrysanthemum plant samples was determined by ICP-MS (Agilent 7500 series, Agilent Technologies, Tokyo, Japan). Samples were pulverized (<100 mesh), digested with HNO_3_-HF (3:1) in a microwave system (Mars 6, CEM), diluted to 50 mL with 2% HNO_3_, and filtered (0.45 µm) prior to analysis, with detection limits of 0.03 and 0.0001. All sample analyses were strictly conducted in compliance with the standards [21,22], with quality control implemented using GBW series soil-certified reference materials (CRMs). The test results are of acceptable quality.

### 2.4. Evaluation of the Soil Remediation Capacity of Chrysanthemum Plants

Cadmium accumulation and years of remediation of contaminated soil were used in this study to assess the remediation capacity of chrysanthemum plants, and they were calculated as follows:

(1) $$\begin{array}{c} M_{soil}=S_{soil}\times h_{soil}\times \rho_{soil}/1000 \end{array}$$

(2) $$\begin{array}{c} M_{Cd}=\left({Cd}_{soil}-0.3\right)\times M_{soil} \end{array}$$

(3) $$\begin{array}{c} C_{Cd}=M_{c}\times {Cd}_{c}/1000 \end{array}$$

(4) $$\begin{array}{c} T=M_{Cd}/C_{Cd} \end{array}$$

where *M_soil_* is the mass of the soil to be removed (kg); *S_soi_*_l_ is the area of the soil to be removed (cm^2^); *h_soil_* is the depth of the soil to be removed (cm); *ρ_soil_* is the density of the soil (g cm^−3^); *M_Cd_* is the mass of Cd in the soil to be removed (mg); *Cd_soil_* is the Cd accumulation in the soil (mg); 0.3 is the current Chinese soil cadmium pollution control standard (mg kg^−1^); *C_Cd_* is the Cd accumulation of chrysanthemum plants (mg); *M_c_* is the dry weight of chrysanthemum plants (g); *Cd_c_* is the Cd content of chrysanthemum plants (mg kg^−1^); and *T* is the remove period.

### 2.5. Data Analysis

This paper used Microsoft Office Excel 2019 for data reduction and analysis, the statistical software Statistical Product and Service Solutions 25 for statistical analysis, and Origin Pro 2022 for drawing statistical charts.

## 3. Results and Discussion

### 3.1. Cd Cumulative Abilities of 23 Chrysanthemum Cultivars

**Figure 2:**
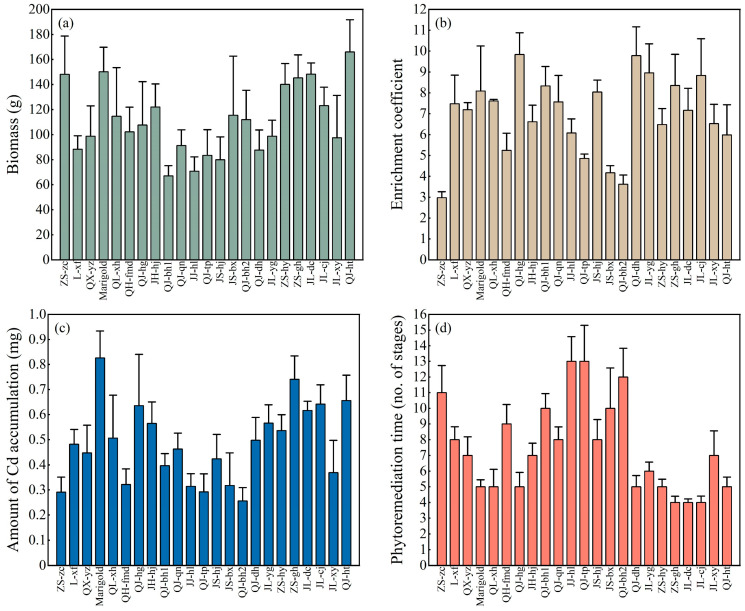
Aboveground biomass (**a**), enrichment coefficient (**b**), Cd accumulation (**c**), and remediation stage (**d**) of 23 chrysanthemum cultivars.

The Cd content of the rhizosphere soil ranged from 0.47 to 0.96 mg kg^−1^, with an average of 0.65 mg kg^−1^. Referring to the current Cd pollution control standard for soil in China (0.3 mg kg^−1^), the exceedance rate of Cd in the soil samples was 100%. Plants absorb heavy metals through the roots and use transport proteins to translocate the metals to aboveground organs for storage. The goal of removing heavy metals in the soil can be achieved by harvesting the plants. Thus, high biomass production is an important factor for harvesting plants to extract greater quantities of heavy metals. All 23 tested chrysanthemum cultivars grew to the full flowering stage in the experimental field. As shown in Figure 2a, the aboveground partial biomass of the tested chrysanthemum cultivars ranged from 67.10 g to 166.08 g, with an average of 111.25 g. A marked difference can be observed between the highest and the lowest values. Among the tested cultivars, the six cultivars with biomass exceeding 140 g were QJ-ht, marigolds, JL-dc, ZS-zc, ZS-gh, and ZS-hy, in that order. The comparison of Cd content between chrysanthemum plants and rhizosphere soil can reflect the Cd absorption capacity of chrysanthemum plants. The aboveground Cd contents of the tested chrysanthemum cultivars ranged from 1.97 to 5.92 mg kg^−1^, with an average of 4.45 mg kg^−1^. As shown in Figure 2b, chrysanthemum plants had a high enrichment capacity for Cd, with enrichment coefficients ranging from 2.98 to 9.84 and averaging up to 6.95. The chrysanthemum cultivars with enrichment coefficients over 8 were, in descending order, QJ-hg, QJ-dh, JL-yg, JL-cj, ZS-gh, QJ-bh1, marigolds, and JS-hj. Under natural conditions in the field, the ability of chrysanthemums in this experiment to accumulate soil heavy metal Cd was higher than reported in high-accumulation plants such as *Arabis paniculata* [20], *Centella Asiatica* [23], *Phytolacca americana* [24], *Potentilla griffithii* [25], *Sigesbeckia orientalis* [26], and *Viola baoshanensis* [27].

The mass of Cd that can be accumulated by each chrysanthemum plant per season is determined by biomass and Cd content [28], and the mass of Cd accumulated by a single chrysanthemum plant in a single season is shown in Figure 2c. The mass of Cd accumulated by the 23 chrysanthemum cultivars ranged from 0.26 to 0.83 mg, with an average of 0.49 mg (Equation (3)). The six chrysanthemum cultivars that accumulated over 0.6 mg Cd were, in descending order, marigold, ZS-gh, QJ-ht, JL-cj, QJ-hg, and JL-dc. Based on the 20 cm chrysanthemum spacing, the average area occupied by each chrysanthemum was 400 cm^2^, h_soil_ was taken as 20 cm, and ρ_soil_ was 1.1 g cm^−3^. As shown in Figure 2d, the remediation of rhizosphere Cd-contaminated soil by the test chrysanthemums required 4–13 stages, with an average of 8 stages (Equations (1), (2), and (4)). There were nine chrysanthemum cultivars with five or fewer remediation stages: ZS-gh, JL-dc, JL-cj, marigolds, QJ-ht, QJ-hg, ZS-hy, QL-xh, and QJ-dh.

The 23 tested chrysanthemum cultivars were compared with domestic and foreign reported Cd hyperaccumulating plants, such as *Portulaca oleracea* [29] and *Sedum alfredii* [30], and a variety of chrysanthemums in this test had obvious Cd hyperaccumulation characteristics and had excellent efficiency in removing soil Cd.

### 3.2. Heavy Metal Tolerance in Multiple-Cropping Chrysanthemum Cultivars

**Figure 3:**
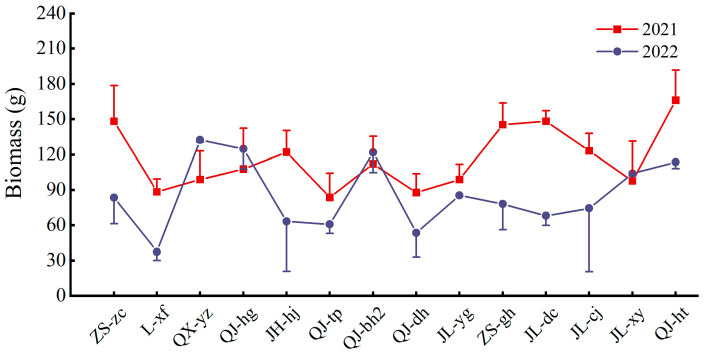
Changes in aboveground biomass of multiple-cropping chrysanthemum cultivars.

Multiple-cropping chrysanthemum cultivars can reduce the cost and increase the efficiency of soil remediation. Fourteen chrysanthemum cultivars grew to full bloom after overwintering in 2022: ZS-zc, L-xf, QX-yz, QJ-hg, JH-hj, QJ-tp, QJ-bh2, QJ-dh, JL-yg, ZS-gh, JL-dc, JL-cj, JL-xy, and QJ-ht. The tolerance of these chrysanthemum cultivars to heavy metals is a prerequisite for their continued use in soil remediation, as shown in Figure 3, which compares the biomass of multiple-cropping chrysanthemums in 2021–2022. The biomass of five chrysanthemum cultivars, QX-yz, QJ-hg, QJ-bh2, JL-xy, and JL-yg, remained stable compared to the previous year, showing that these chrysanthemum cultivars are more tolerant to heavy metals. Most of the multiple-cropping chrysanthemum biomass was lower than that of the previous year, which may be due to the decline in soil fertility or the growth inhibitory effect of the heavy metal Cd on chrysanthemum plants. Based on Cd content in chrysanthemum plants in 2021, the remediation of rhizosphere soils should be extended by an average of 3.29 stages.

### 3.3. Enrichment Coefficient to Different Aboveground Parts of Multiple-Cropping Chrysanthemum Cultivars

**Figure 4:**
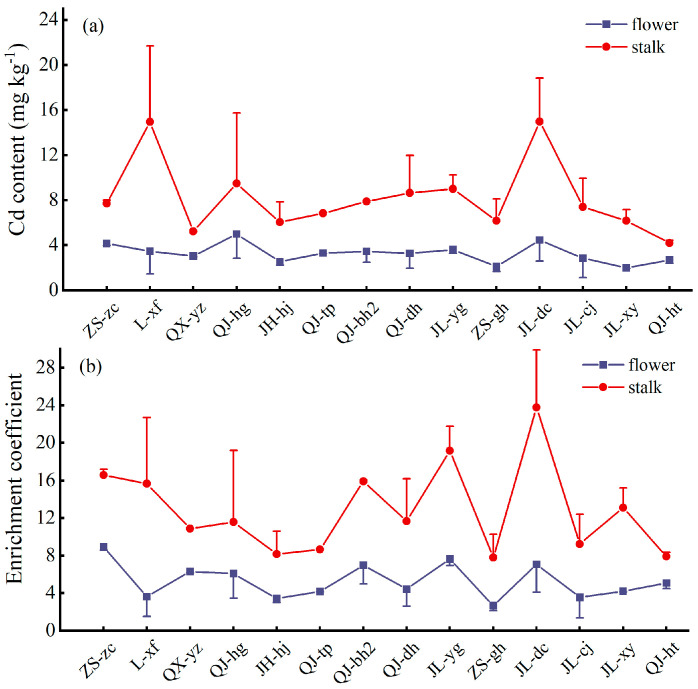
The Cd content (**a**) and enrichment coefficient (**b**) in flowers and stalks.

The mechanism of heavy metal absorption may differ among plant cultivars growing in heavy metal-contaminated soil. Some toxic heavy metals are fixed in the root, whereas other cultivars tend to transport the heavy metal to the aboveground parts [31]. The xylem vessels play an important role in the long-distance transport of solutes from the roots, and some toxic heavy metals in the roots can bypass the phloem to enter the xylem and are then transported to aboveground organs [32]. Previous studies have reported that the relative Cd accumulation factors for different organs of chrysanthemums were ranked as follows: leaf > stem > flower [33]. In the present study, 14 multiple-cropping chrysanthemums were analyzed for the content and enrichment coefficient of Cd in flowers and stalk, as shown in Figure 4. The Cd content in the flower of 14 chrysanthemum cultivars was distributed between 1.98 and 4.98 mg kg^−1^, with an average of 3.28 mg kg^−1^, and the enrichment coefficients were distributed between 2.67 and 8.94, with an average of 5.30. The Cd content of the stalk was distributed between 4.20 and 15.00 mg kg^−1^ with an average of 8.19 mg kg^−1^, and the enrichment coefficients were distributed between 7.80 and 23.77, with an average of 12.86. The Cd content and enrichment coefficients of all tested chrysanthemum stalks were higher than those of flowers, and chrysanthemum plant stalks were superior to flowers, which showed that chrysanthemum stalks accounted for the major portion of the whole chrysanthemum plants in terms of Cd accumulation.

### 3.4. Cd Enrichment Efficiency of Chrysanthemum Plants Under Organic Acid and EDTA-Si

**Figure 5:**
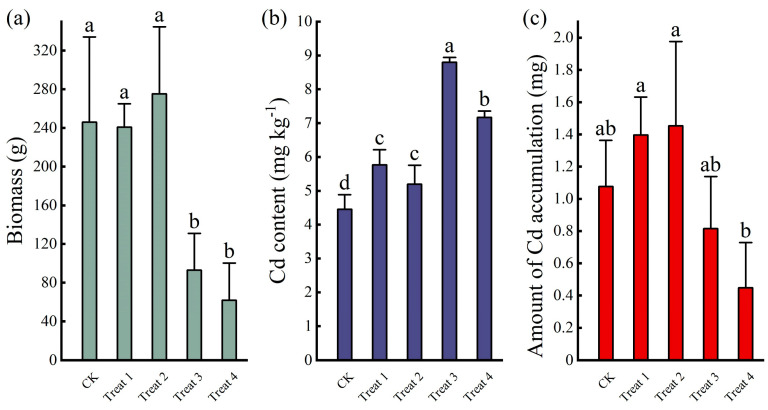
Aboveground biomass (**a**), Cd content (**b**), and Cd accumulation (**c**) of chrysanthemum plants under organic acid and EDTA-Si. Note: Lowercase letters a,b,c,d above the error bar represents the significant differences (*p* < 0.05) among different treatments, these containing the same letters show no significant difference (i.e., ab and a), while those with different letters indicate a significant difference between the two (i.e., a and b).

The QX-yz cultivar with stable biomass and high Cd accumulation was subjected to bamboo vinegar and EDTA-Si application tests, as shown in Figure 5. There was no significant change (*p* < 0.05) in chrysanthemum biomass in treatments 1 and 2 compared to the control, which showed that the concentration of bamboo vinegar used in the experiment did not have a toxic effect on chrysanthemum growth. The Cd content of chrysanthemum plants in treatments 1 and 2 was significantly increased by 29.52 and 16.65 percentage points (*p* < 0.05) compared with the control, which indicated that the bamboo vinegar promoted Cd^2+^ accumulation through the roots of chrysanthemum plants, and the formation of Cd^2+^ complexes and the decrease in soil pH led to the increase in the Cd content of the exchangeable state in the soil. Cd accumulation in chrysanthemum plants was elevated by 29.63 and 34.99 percentage points in treatments 1 and 2 compared with the control. Treatment 3 chrysanthemum biomass significantly decreased by 62.22 percentage points (*p* < 0.05) compared to the control; treatment 4 significantly decreased by 74.85 and 77.53 percentage points (*p* < 0.05) compared to the control and treatment 2, which showed that the growth of chrysanthemum plants was inhibited. Similarly, the toxic effect of EDTA on plants was observed in the enrichment study of heavy metals in *Brassica juncea* [34]. Studies have shown that high levels of Cd are toxic to chrysanthemum plants, and in addition, EDTA may complex Ca^2+^ and Zn^2+^ to disrupt the biofilm, causing damage to chrysanthemum plants during the growth period [35]. Since the chelation of EDTA-Si with Cd^2+^ in the soil formed a stable water-soluble complex, the Cd content of chrysanthemum plants in treatments 3 and 4 significantly increased by 97.53 and 61.02 percentage points (*p* < 0.05). A comparison of treatment 2 with treatment 3 showed that the biological activity of Cd under EDTA-Si treatment was higher than that of bamboo vinegar (*p* < 0.05). However, the reduced biomass of chrysanthemum plants under EDTA-Si treatment caused a decrease in Cd accumulation in chrysanthemum plants in treatments 3 and 4.

Bamboo vinegar solution and EDTA-Si could significantly promote the accumulation of Cd in chrysanthemum plants in this experiment; in addition, the use of EDTA-Si caused a significant decrease in the biomass of chrysanthemums; however, the effects on chrysanthemum plants at different application rates were not clear. Although treatments 1 and 2 used different concentrations of bamboo vinegar, they did not differentiate in the amount applied, and no significant changes in chrysanthemum biomass and Cd content were observed (*p* < 0.05). Therefore, the rational application of bamboo vinegar and EDTA-Si to chrysanthemum plants needs to be further investigated. As far as the results are concerned, treatments 1 and 2 better improved the Cd accumulation efficiency of chrysanthemum plants, and under the premise of constant biomass, the remediation of contaminated soil by QX-yz cultivars after applying bamboo vinegar could be advanced by 1 year, which can be used for the remediation of contaminated soil by popularizing the use of chrysanthemum plants.

### 3.5. Estimation of the Economic Benefits of Chrysanthemum Remediation for Cd-Contaminated Agricultural Land

The remediation of heavy metal-contaminated soil through engineering excavation methods often requires a long time and high costs [36]. Moreover, during the remediation process, the contaminated farmland generates no economic returns. Although phytoremediation has received increasing attention in recent decades as an environmentally friendly and cost-effective green technology, its practical application remains limited. This is primarily due to the slow growth and low biomass of remediating plants, resulting in low remediation efficiency, and the long duration typically required for the remediation of heavy metal-contaminated farmland. A more critical issue is that most hyperaccumulator plants for heavy metals are non-economic species, meaning that the long-term remediation process of contaminated farmland does not generate income but requires significant investment [37]. To promote the practical application of phytoremediation, it is necessary to establish an effective and economically beneficial phytoremediation system. This would enable owners of contaminated farmland to earn annual economic returns during the phytoremediation of contaminated soil.

In this study, the selected chrysanthemum varieties for remediation can be continuously harvested and sold during the remediation process of contaminated soil, generating economic benefits. If we consider only the input costs and revenue from chrysanthemum sales, without considering the environmental and social benefits of contaminated soil remediation, we have calculated a detailed economic account.

To enhance the effectiveness of phytoremediation in removing heavy metals from the soil, it is necessary to construct some auxiliary planting facilities to ensure double cropping of chrysanthemums and maximize their biomass. The construction and maintenance costs of chrysanthemum planting facilities are 0.75 × 10^4^ RMB per hectare per year, while the costs of fertilizers and pesticides for chrysanthemum cultivation are 3.15 × 10^4^ RMB per hectare per year, and the costs of machinery and tools are 0.525 × 10^4^ RMB per hectare per year. Additionally, land rental costs are 1.8 × 10^4^ RMB per hectare per year.

The average yield of chrysanthemum branches per hectare of contaminated farmland is 3.89 × 10^6^ branches (Table 3). Most chrysanthemums are sold as ceremonial items to funeral homes across Zhejiang Province. The price of chrysanthemums varies with time and quality. Generally, ordinary-grade chrysanthemums account for 80%, with each bundle (14 branches) selling for 2.5 RMB; high-grade chrysanthemums account for 5%, with each bundle selling for 5 RMB; and low-grade white chrysanthemums account for 15%, with each bundle selling for 1 RMB.

A farmer can plant chrysanthemums on 0.1 hectares of land and harvest two crops per year. After deducting all the aforementioned costs, the farmer’s net income from chrysanthemum cultivation is 6.17 × 10^4^ RMB, which is higher than the average annual income of residents in Yongkang City, Zhejiang Province, which is 5.67 × 10^4^ RMB.

This indicates that by planting chrysanthemums for the remediation of heavy metal-contaminated soil, there is no need to pay for land clearing costs and land idleness losses, and economic benefits can be earned through the sale of chrysanthemums. The net income from chrysanthemum cultivation in this study is higher than that generated by the phytoremediation of heavy metal-contaminated soil using marigolds [38]. In conclusion, the selected dominant chrysanthemum varieties exhibit vigorous growth, high biomass, strong tolerance to toxic heavy metals, and demonstrate strong Cd accumulation and removal effects. This study primarily focuses on the accumulation and removal effects of chrysanthemums on Cd, and they may have similar effects on other harmful heavy metals. More importantly, planting white chrysanthemums for phytoremediation can also generate considerable economic benefits.

## 4. Conclusions

In this study, 23 chrysanthemum cultivars were tested. Six Cd hyperaccumulating chrysanthemum cultivars were marigolds, ZS-gh, QJ-ht, JL-cj, QJ-hg, and JL-dc, the average Cd accumulation per plant was over 0.6 mg, and their remediation of rhizosphere soils required 4–5 years. There are 14 multiple-cropping cultivars, of which the biomass of chrysanthemum cultivars QX-yz, QJ-hg, QJ-bh2, JL-xy, and JL-yg remain stable. The highest Cd accumulation was 0.64 mg in the QJ-hg single plant. The use of EDTA-Si can bring about toxic effects and cause chrysanthemum biomass to decline. Bamboo vinegar is more helpful to increase the Cd accumulation of chrysanthemum plants. In this experiment, the application of bamboo vinegar led to Cd accumulation within chrysanthemums being elevated by up to 34.99 percentage points, and the number of stages of soil remediation was expected to be reduced by 1. Additionally, chrysanthemum sales provide sustainable economic benefits to the phytoremediation process, with practitioners in chrysanthemum cultivation earning above the local residents’ average annual income level.

## Figures and Tables

**Table 1:** The full cultivar names of chrysanthemums.

Code	Variety	Code	Variety	Code	Variety
ZS-zc	Zhongshanzichen	QJ-bh1	Qingjianbaihuang1	JL-yg	Jinlingyuegui
L-xf	Lüxuanfeng	QJ-qn	Qingjianqingniao	ZS-hy	Zhongshanhongyun
QX-yz	Qianxiuyinzhen	JJ-hl	Jinjihongling	ZS-gh	Zhongshanguanghui
Marigold	Marigold	QJ-tp	Qingjiantaiping	JL-dc	Jinlingdianchun
QL-xh	Qunlongxihai	JS-hj	Jinsihuangju	JL-cj	Jinlingcuiju
QH-fmd	Qinhuaifenmudan	JS-bx	Jinsibaoxiao	JL-xy	Jinlingxiaoye
QJ-hg	Qingjianhuanggai	QJ-bh2	Qingjianbaihuang2	QJ-ht	Qingjianhuangtianzan
JH-hj	Jiuhuahongjing	QJ-dh	Qingjiandahuang		

**Table 2:** Experimental design to test the effects of bamboo vinegar and EDTA-Si application on Cd accumulation.

	Preparation	Application
Control (CK)	Purified water	1000 mL
Treatment 1	Diluted 15 mL of bamboo vinegar with purified water to 4500 mL	1500 mL
Treatment 2	Diluted 15 mL of bamboo vinegar with purified water to 3000 mL	1000 mL
Treatment 3	Dissolved 15 mg EDTA-Si in purified water and diluted it to 3000 mL	1000 mL
Treatment 4	Dissolved 15 mL of bamboo vinegar and 15 mg EDTA-Si in purified water and diluted it to 6000 mL	2000 mL

Note: EDTA-Si is a silica-immobilized EDTA composite.

**Table 3:** Annual yield of chrysanthemum flowers in the contaminated farmland and theoretical annual income per hectare.

Chrysanthemum Varieties	Flower Count (hm^−2^ year^−1^)	Net Income (RMB hm^−2^ year^−1^)
QX-yz	3.96 × 10^6^ ± 0.42 × 10^6^	61.67 × 10^4^ ± 9.26 × 10^4^
QJ-hg	3.89 × 10^6^ ± 0.72 × 10^6^	60.46 × 10^4^ ± 8.38 × 10^4^
QJ-bh2	4.02 × 10^6^ ± 0.86 × 10^6^	62.69 × 10^4^ ± 11.34 × 10^4^
JL-xy	3.69 × 10^6^ ± 0.41 × 10^6^	57.03 × 10^4^ ± 7.87 × 10^4^
JL-yg	4.16 × 10^6^ ± 0.58 × 10^6^	65.09 × 10^4^ ± 10.43 × 10^4^
Average	5.31 × 10^6^ ± 1.00 × 10^6^	60.46 × 10^4^ ± 8.63 × 10^4^
Marigold		16.88 × 10^4^~55.70 × 10^4^

## Data Availability

Data will be made available upon request.
